# Analysis of Surface Characteristics of ProTaper Universal and ProTaper Next Instruments by Scanning Electron Microscopy

**DOI:** 10.4317/jced.54049

**Published:** 2017-07-01

**Authors:** Jeffery Bennett, Kwok-Hung Chung, Hanson Fong, James Johnson, Avina Paranjpe

**Affiliations:** 1 Department of Endodontics, University of Washington, Seattle; 2 Department of Restorative Dentistry, University of Washington, Seattle; 3 Department of Material Science and Engineering, University of Washington, Seattle

## Abstract

**Background:**

Many new rotary files systems have been introduced, however, limited research has been conducted related to the surface irregularities of these files and if these have any effects on the files themselves. Hence, the aim of the present study was to analyze surface irregularities of the ProTaper® Universal rotary files (PTU) and the ProTaper Next™ rotary files (PTN) before and after instrumentation in curved canals. The main objective was to investigate the nature of these irregularities and how they might influence the use and fracture of rotary files during root-canal treatments.

**Material and Methods:**

The files were examined pre-operatively using a stereomicroscope and scanning electron microscopy(SEM) to analyze surface imperfections and the presence of particles. Mesial roots of forty extracted mandibular molars were selected. Each instrument was used to prepare one of the mesial canals. The files were then rinsed with alcohol, and autoclaved and analyzed again.

**Results:**

Of the 80 files used in this study, five files fractured, five files unwound and seven files were curved or bent and they all belonged to the PTU group. Irregularities and debris could be visualized with the SEM on both unused PTU and PTN files. Most of the debris was found associated with deeper milling grooves and defects on the surface of the metal. Surface analysis of the files that were used and sterilized were performed and the SEM images demonstrated organic debris, metal flash, and crack formation and initiation of fractures for both file types. All files showed machining grooves, metal flash, debris, and defects on cutting edges.

**Conclusions:**

These irregularities appear to be critical in the accumulation of debris and initiation of fatigue and crack propagation within the NiTi alloy. The accumulation of debris could be a concern due to the potential exchange of organic debris between patients.

** Key words:**ProTaper® Universal, ProTaper Next™, surface characteristics, SEM.

## Introduction

Nickel-titanium (NiTi) was developed in the early 1960’s and Walia *et al.* first introduced hand files for endodontic use in 1988 (1). The files were observed to be more flexible compared to stainless steel files and superior in resistance to torsional fracture (1). The flexibility of nitinol can be attributed to a low modulus of elasticity compared to stainless steel, while the superior fracture resistance is due to the ductility of the alloy (1). NiTi rotary files have become increasingly popular and have proven to be safe, efficient, and well adaptive to shape even severely curved root canals (2).

Though these traditional NiTi files have been popular, manufacturers have tried various methods to improve the performance of NiTi in order to make them more efficient and more resistant to deformation and fracture. In the past years there have been significant enhancements in the design and control of the raw materials relative to microstructure, material properties, and manufacturing processes for endodontic instruments (3). One approach that has been implemented includes altering the cross-sectional shape of the wire blanks. The cross sectional design of the files has been shown to alter the mechanical properties of rotary files (4). Other methods include producing better NiTi alloys through heat treatment and M-wire technology (Dentsply Tulsa Dental Specialties, Tulsa, OK). M-wire technology implements a thermo-mechanical processing procedure that produces a super-elastic wire blank that has improved mechanical properties compared with traditional austenitic NiTi wires (5). Johnson et al. examined M-wire files and reported that there was a nearly 400% increase in resistance to cyclic fatigue compared to that of traditional NiTi austenitic wires of the same type and shape (6). Recently, the manufacturer of the ProTaper® Universal (Dentsply Tulsa Dental Specialties, Tulsa, OK) (PTU) file system attempted to improve on the mechanical properties and performance of the popular system by utilizing M-wire technology, implementing different tip sizes and tapers, and using a rectangular cross-sectional geometry with the ProTaper Next™ file series (Dentsply Tulsa Dental Specialties, Tulsa, OK) (PTN).

The manufacturing of these NiTi rotary files is a complex procedure, and it has been that this could result in a high concentration of defects such as debris and metal strips, in addition to pits and blunt cutting edges (7). Other studies have demonstrated a change in the surface of rotary instruments after use (8). Although many previous studies have investigated various aspects of the ProTaper Next™ and ProTaper® Universal files, which include canal shaping ability (9), cyclic fatigue resistance (10), amount of debris extruded (11) etc., no studies have investigated how the surface and the alloy features would affect instrument fracture. Hence, the aim of this study was to analyze surface irregularities of these two rotary files before use and after instrumentation in curved canals in extracted human teeth. The main objective was to investigate the nature of these irregularities and how they might influence the use and fracture of these rotary files during root-canal treatment.

## Material and Methods

-Selection of teeth.

Forty extracted mandibular molars in which the mesiobuccal (MB) and mesiolingual (ML) canals had separate foramina were initially selected for use in this study. The teeth were radiographed and the initial root curvatures were determined as previously stated by Pruett *et al.* (12). Teeth with curvatures between 60 and 90 degrees and 2 to 5 mm radius of curvature were selected.

-Preoperative examination of the rotary files.

**Figure 1:**
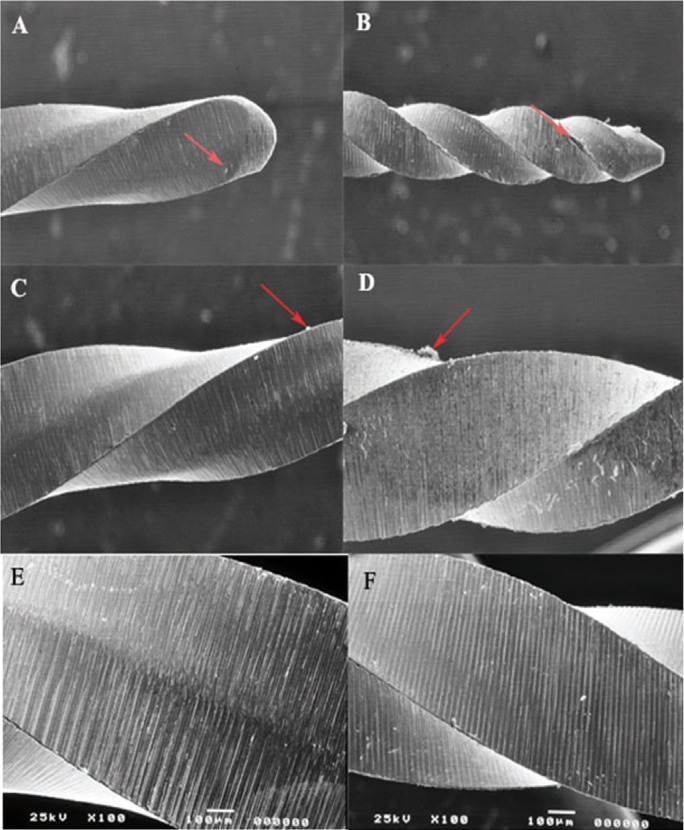
SEM images of new files.SEM images of new PTU and PTN rotary files debris was seen on all new files of PTU (A and C) and on PTN files (B and D) as indicated by the red arrows. Images A and B were taken at 20X magnification and images C and D were taken at 100X magnification. Milling grooves are apparent on PTU files (E) and PTN files (F).

**Figure 2:**
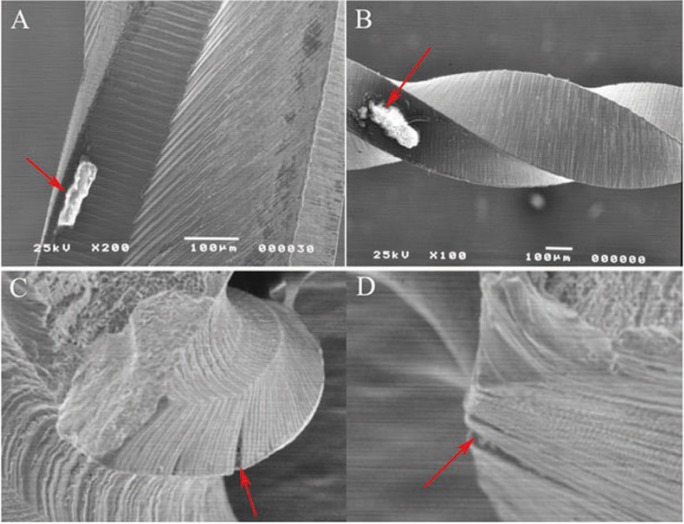
SEM images of used and sterilized PTU and PTN rotary files. Debris was seen both file types PTU (A) and PTN (B). Figures 2C and 2D show crack formation and initiation of fractures in the PTU and PTN respectively.

**Figure 3:**
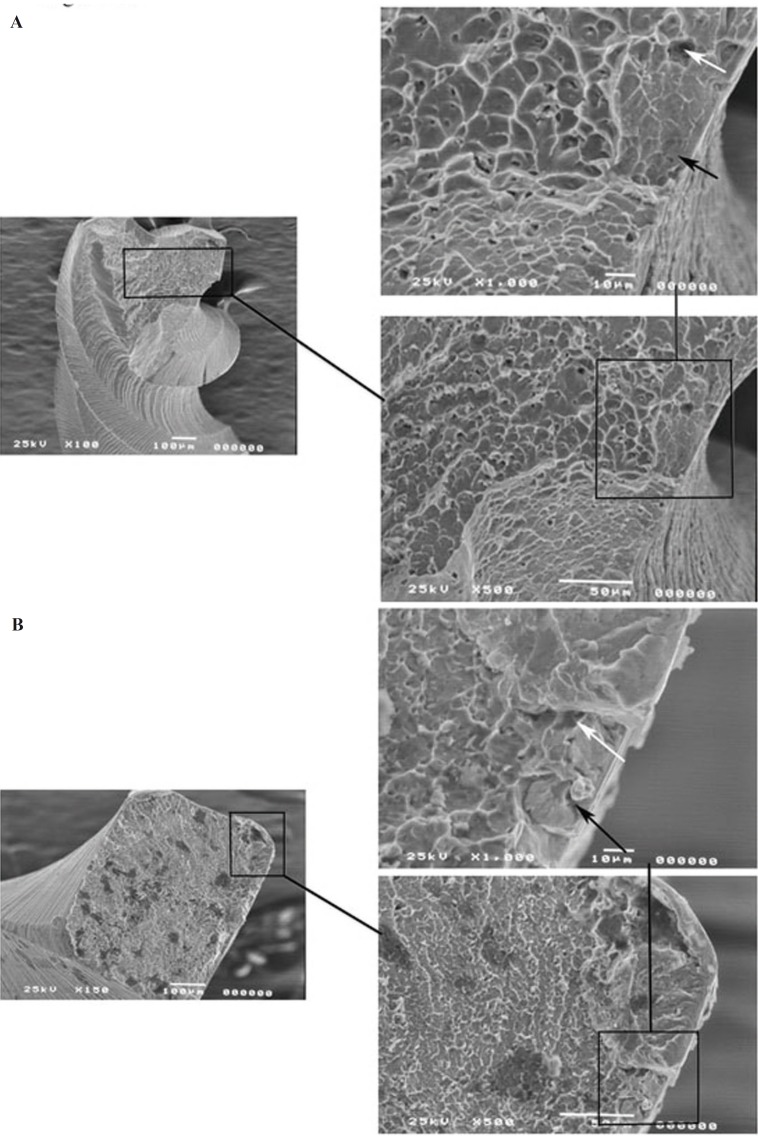
SEM images of files fractured by cyclic fatigue. Figure 3A is an SEM image of a PTU F3 file fractured and Figure 3B is an SEM image of a PTN X3 file fractured by cyclic fatigue. The sub-figures show a magnified image of one part of the fracture surface which demonstrates the beginning of the fracture at this inclusion (black arrows) where it progresses to voids within the body of the alloy (white arrows). Then past this point we can see a fast fracture under load, represented by the signs of ductile fracture.

**Figure 4:**
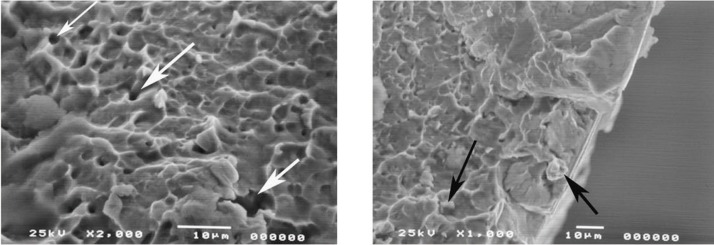
A representative image of the fractured surface of a file PTN X3. The figure demonstrates micro-voids (white arrows) and inclusions (black arrow) of varying sizes that were also seen distributed throughout the fracture surfaces of the files.

A total of 80 rotary instruments, 40 ProTaper® Universal and 40 ProTaper Next™ rotary files were selected. They were with-drawn from sealed boxes and sequentially numbered on the handle, using a high-speed diamond bur. The files were then inspected with a stereomicroscope (SMZ 1500, Nikon Inc., Melville, NY), at magnifications between 20X and 100X. Configurative changes, surface imperfections, and the presence of particles were observed, using an endodontic ruler, to establish the position of the surface irregularities in relation to the instrument tip. Each instrument was provided with an identification record, used to guide the analysis by scanning electron microscopy (SEM) (FEI Sirion FEG microscope Hillsboro, OR). Scanning electron images showing the topographic characteristics of the irregularities and debris, were captured. Surface morphology of the specimens were examined at a magnification of 100-250X. Images of 100µm areas were taken and representative images are shown in the figures 1-4.

-Instrumentation

Following this initial analysis, the canals were randomly assigned into two groups: 40 canals to Group U – ProTaper® Universal (PTU) files S1, S2, F1, F2, F3 and 40 canals to Group N – ProTaper Next™ (PTN) files X1, X2, X3. The coronal accesses for the selected teeth were completed, and the chambers were irrigated with 6% sodium hypochlorite (NaOCl). The working length of each canal was determined by using a #10 K-File (Dentsply, Tulsa Dental Specialties, OK, USA). The files were inserted into the canals until visually seen past the apical foramen then backed up 1mm. A reproducible glide path was created using the 10 K-file. A single operator who was competent in both instrumentation techniques performed the instrumentation. New files were used for each instrumented canal.

Each instrument was used to prepare one of the mesial root canals with an average curvature between 60 and 90 degrees and 2 to 5 mm radius of curvature according to Pruett et al. (12). Instrumentation was performed according to manufacturer instructions. Group U teeth were instrumented to F3, while Group N teeth were instrumented to X3. Each file was used with a DTC rotary handpiece at 300 RPM and 200 g-c (Dentsply Tulsa Dental Specialties, OK, USA). The files were then cleaned in an ultrasonic cleaning unit (BioSonic UC125, Coltène/Whaledent Inc. Cuyahoga Falls, OH) for 15 min with detergent, rinsed with alcohol, and autoclaved (Statim 2000, SciCan Inc., Canonsburg, PA) after canal preparation.

-Post-operative examination of the rotary files.

These used rotary files were then examined by stereomicroscope and SEM as described above in the pre-operative examination section to analyze defects and the presence of debris.

## Results

Of the 80 files used in this study, a total of 5 files fractured (2 F2 and 3 F3), 5 files unwound (1 F2 and 4 F3), and 7 files were curved or bent (3 F2 and 4 F3) and all these files belonged to the PTU group. Distribution of the files is summarized in  Table 1.

**Table 1:**
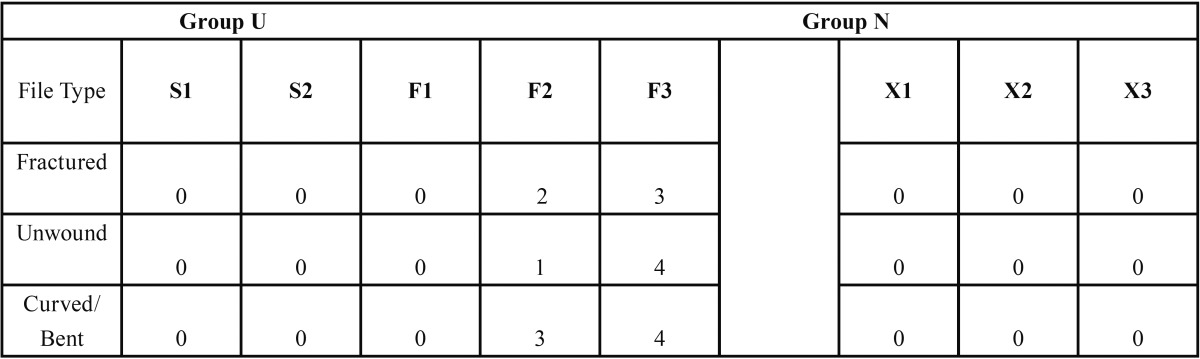
Number of files with gross defects and fracture in each group.

Morphometric changes such as the presence of machining grooves, metal flash, and debris could be visualized by stereomicroscopy and SEM in all the new files. Figure 1 is a representative image of the irregularities and debris in unused files as seen under the SEM. Figures 1A &C show the debris present on new PTU files and figures 1B &D demonstrated debris on new PTN files. The irregularities and debris were distributed randomly along the length of all files and most of these findings were only visible when viewed with SEM. The exact location, distribution, size and thickness of the material varied from one instrument to another, irrespective of size and taper. Most of the debris was found associated with deeper milling grooves and defects on the surface of the metal as seen in figure 1E (PTU) and 1F (PTN).

Surface analysis of the files that were subjected to instrumentation and sterilization procedures, was performed by stereomicroscopy. The files appeared to have minimal morphological changes except the files that were bent, unwound, or fractured (data not shown). However, the SEM images showed a variety of differences in the files after they had been used in the extracted teeth. Figure 2 shows the SEM images demonstrating organic debris, metal flash, and crack formation and initiation of fractures for both PTU (Figures 2A &C) and PTN (Figures 2B &D). Crack origins were typically seen associated with manufacturing defects near the surface of the metal. Figures 2C and 2D are representative images of a crack initiation sites for both files.

Files from both groups (F3 and X3 files) were subjected to fractures due to cyclic fatigue as described previously (13). The fractured files examined by stereomicroscopy showed signs of both torsional and flexural fracture, according to classification by Sattapan *et al.* (14). However, when these fractured surfaces were examined under the SEM, they demonstrated a ductile fracture associated with a defect within the file. Figures 3A and B are views of the fractured surfaces of an F3 PTU and an X3 PTN file respectively. The sub-figures show a magnified image of one part of the fracture surface. As seen from the figure both file types showed the beginning of the fracture at the inclusions (black arrows) from where it progressed to voids (white arrows) within the body of the alloy. The start of the ductile fracture was associated with larger voids or fractures within the metal. Figure 4 is another representative image of the fractured surface of a file PTN X3 demonstrating inclusions and microvoids of varying sizes that were also seen distributed throughout the fractured surfaces of the files. Similar voids and inclusions were seen in the PTU F3 file group.

## Discussion

Newer rotary NiTi instruments have been introduced which have increased flexibility and strength, however, they are still vulnerable to fracture. Fractures usually are either ductile or brittle fractures. Ductile fractures are caused by slow tearing of the material, displaying a dimpled surface created by growth of internal voids of the metal and an appreciable gross plastic deformation (15). Hence, manufacturers are constantly attempting to improve these instruments. One such improvement is the introduction of files made with M-wire. M-wire is expected to have a higher strength and wear resistance compared to conventional austenitic NiTi wire because of its martensitic structure (16). The grain size of M-wire is smaller than conventional NiTi wires. Ye *et al.*, (16) proposed the 100 nm martensite grains in M-Wire contributed to the improved torque strength and wear resistance. Smaller grain sizes of the alloy can reduce crack initiation along grain boundaries caused by locally concentrated stress, therefore increasing the yield strength of the alloy (17). This may be part of the explanation of why none of the PTN files fractured in this study ( Table 1). However, no study till date has looked at the surface irregularities of files made with M-wire and how these affected fracture. Hence, this study compared two different file systems namely ProTaper® Universal which uses traditional NiTi and ProTaper Next™ which uses the M-wire technology.

Scanning Electron Microscopy (SEM) was used in this study to examine the fractured surface of rotary instruments. This is becoming a more common practice for fractographic examination of rotary instruments (18-22). Many studies previously examined fractured files in a lateral view with lower power microscopy and low power SEM (14,23). Lateral inspection of ProFile rotary files according to Sattapan *et al.* (14) were classified as being 56% due to torsional and 44% as flexural fatigue. Findings from this present study showed that files appearing as torsional fatigue from a lateral view from stereomicroscopy and SEM indeed had features of both torsional and flexural fatigue when the fracture surface was examined. Fatigue was represented by characteristic striations seen on the fractured surface of some of the files (data not shown). In contrast Sattapan *et al.* (14) and Cheung *et al.* (19) demonstrated that two-thirds of the separated instruments failed due to fatigue revealed by striations on the fractured surface. Further, Wei *et al.* (24) found that 88% of fractured files failed after clinical use as a result of flexural fatigue. These findings clearly illustrate the clinical importance of both fatigue and torsional stress in the fracture of endodontic rotary files. One inconsistency between many studies, particularly clinical studies with fractured instruments, is the number of times the files were used before fracture. New files of each type were examined by stereomicroscopy and SEM. All files showed the presence of irregularities and defects as seen in figure 1. All files showed varying amounts of machining grooves, metal flash, debris, and defects on cutting edges. These findings were consistent with other studies examining the surface of new files by SEM (25). The presence of surface defects appeared to be very important in the initiation of the fracture process of the used files. Fractographic analysis showed the presence of these defects to be associated with the initiation site of fatigue in all the files that fractured in the extracted teeth (Fig. 2). These defects act as weak points where stress is concentrated in the file, leaving the alloy more susceptible to fatigue and ultimately leading to crack propagation as stress continues (19). Next, one file from each group was made to fracture due to cyclic fatigue. An example of a PTU F3 file fractured by cyclic fatigue can be seen in figure 3A and the PTN X3 can be seen in figure 3B. Both figures demonstrate the beginning of the fracture at the inclusion where it progresses to voids within the body of the alloy. Then past this point is a fast fracture under load, represented by the signs of ductile fracture. Notable there were presence of small voids distributed throughout the bulk of the material within the body of the alloy (Fig. 3A,B). These findings were also observed in other studies (21). Porosities or voids could be visualized on the fractured surfaces of the files (Fig. 4). Porosities in nickel- titanium compacts can be from different sources including the Kirkendall effect, alloying effect, and a liquid capillary effect (26). Ounsi *et al.* (21) proposed that these voids were due to the Kirkendall effect. The Kirkendall effect is where the diffusion of nickel atoms into titanium is much faster than that of titanium atoms in the opposite direction, creating unbalanced mass transports leading to the formation of voids in the original nickel side of the alloy (26). Pirani *et al.* proposed that the voids might be caused by inclusions within the alloy grains and grain boundaries that may fracture or debond under an applied load (22).

The inclusions within NiTi alloy are assumed to be titanium carbonitrides and NiTi oxides formed during the vacuum melting and casting in graphite cubicles (22,27) (Fig. 4). This process causes slight carbon contamination in the melt, forming the oxide impurities that appear evenly distributed within the NiTi matrix (27). The concentration and distribution of the inclusions has been proposed as a method of assessing the metallurgical quality of the NiTi alloy produced by different manufacturers of rotary instruments (28). However, there was no apparent difference in the distribution of inclusions between PTU and PTN rotary files. The voids and inclusions were distributed evenly within dimples associated with ductile fracture in all samples (Fig. 3). This is an important observation as brittle fractures are highly undesirable. Unlike brittle or quasi-brittle fracture, ductile fracture initiates with a longer period of stable crack or void growth due to plastic deformation. In this type of fracture, the microvoids present in the alloy nucleate at the interface between the matrix and secondary phase particles (29). Therefore, these inclusions and micro-voids present within the alloy due to the manufacturing process appear to be very important in the fracture initiation and propagation in NiTi rotary files. It can be inferred that improvements in the fabrication process of the alloy to reduce these irregularities may increase the resistance to fracture and fatigue of these instruments.

An interesting finding was the frequency of the presence of remaining organic debris on the surface of the rotary files even after thorough ultrasonic cleaning and autoclaving (Fig. 2A,B). This finding was in accordance with previous studies where dentin debris was found adhering to the file surface after use and cleaning (18,20,30). It is suspected that the organic debris adheres to and remains on the file due to mechanical retention within irregularities both from manufacturing flaws and defects created during instrumentation. It can be assumed that these dentinal chips and other debris from root canal preparation *in vivo* will remain after extensive ultrasonic cleaning and autoclaving. This remaining debris has implications that should be studied further. Alapati *et al.* proposed that embedded dentin deposits cause a wedging action within machining cracks and may lead to their propagation resulting in instrument distortion and failure (18). Another concern is the potential exchange of organic debris between patients. This debris is assumed to be sterile after autoclaving, but its presence may be a source of antigens, infecting agents, or nonspecific irritants (31). Another study has demonstrated that prions can be present in human dental pulp tissue and there may be a risk of infection by contaminated files (32). However, research is lacking on the risk of infection and further studies should be pursued before a recommendation can be made.

## Conclusions

Within the conditions of this study, new files of each type (PTU and PTN) examined by stereomicroscopy and SEM all showed the presence of irregularities and defects. All files showed machining grooves, metal flash, debris, and defects on cutting edges. These irregularities appear to be critical in the accumulation of debris and in the initiation of fatigue and crack propagation within the NiTi alloy. All files showed some degree of metal fatigue near alloy irregularities with ultimate failure resulting in ductile fracture. The fractured files observed with SEM showed varying degrees of inclusions, microvoids, signs of dimpled rupture, surface cracks, and organic debris.
